# Recognizing and Adapting to Cultural Differences: Influence of International Educational Programs on Future Nursing and Midwifery Practice

**DOI:** 10.1177/10436596231198276

**Published:** 2023-09-22

**Authors:** Jacqueline Johnston, Lisa McKenna, Gulzar Malik, Sonia Reisenhofer

**Affiliations:** 1 La Trobe University, Melbourne, Victoria, Australia; 2 Bairnsdale Regional Health Service, Victoria, Australia

**Keywords:** international, education, nurse, midwife, grounded theory, culture

## Abstract

**Introduction:**

International educational programs build cultural humility and safety skills in nursing and midwifery students; however, long-term outcomes of these programs are unclear. The purpose of this study was to explore the impact of international educational programs on nurses’ and midwives’ future professional practice.

**Method:**

Using grounded theory informed by Charmaz, 13 general nurses, two mental health nurses, three midwives, and four dual-qualified nurse/midwives across eight different countries were interviewed. Three categories evolved from the analysis. This article reports on the category *Recognizing and adapting to cultural differences.*

**Findings:**

Participants developed cultural safety and awareness from participation in programs extending into future practice. Experiencing and adapting to cultural similarities and differences, they developed culturally congruent practices many years after program completion.

**Discussion:**

International programs contributed to participants’ professional practice. Positive and ongoing influences are important for employers to promote patient safety and culturally congruent quality care. Findings are also relevant for education providers to inform quality cultural learning.

## Introduction

Essential skills of cultural humility and global citizenship attributes, such as critical thinking and effective teamwork, are required for nursing and midwifery graduates to provide quality care to increasingly diverse populations (Foronda et al., 2016; Villar-Onrubia & Brinder, 2016). Students need opportunities to develop their cultural competency in preparation for professional practice. One strategy for achieving this is for education institutions to offer international educational programs such as semester exchange, short-term study abroad, and service-learning where students travel to different countries to experience cultures different to their own (Karnjuš et al., 2020).

Participation in an international educational program during a nurse’s or midwife’s pre-registration education has been shown to have many short-term benefits such as increased cultural awareness and competence (Granel et al., 2021). However, a recent review (Johnston et al., 2022) demonstrated a lack of reported longer term impact/influence and indicated a need to investigate long-term outcomes of international educational programs on students’ future cultural competence and professional practice.

## Background

Upon graduation, nurses and midwives are expected to be culturally competent global citizens ready to deliver health services to people from diverse cultural backgrounds and ethnicities (Gower et al., 2016). However, Cuellar (2016) notes that most undergraduate students have limited education related to cultural beliefs and health practices. One way to enable nursing and midwifery pre-registration students to have cultural learning experiences is for institutions to offer international educational programs, whereby students undertake part of their studies in a different culture, either within their own country or overseas. The literature (Cuellar, 2016; Granel et al., 2021; Sedgwick & Atthill, 2020) asserts that offering such opportunities is a necessary step to help nursing graduates successfully work with populations from culturally diverse backgrounds.

As programs range in their aims, duration, and presence or not of clinical experiences (Browne et al., 2015), terminology used in the literature to describe them differs among countries and institutions. “Service-learning” programs feature predominately in American and Canadian literature and are described as course-based, credit-bearing educational experiences offering students opportunities to improve their cultural awareness (Matthew et al., 2018; Sedgwick & Atthill, 2020). “Exchange” describes programs undertaken within a student’s official education program, usually of a 6- to 12-month duration, and often involving reciprocal agreements between universities (Tjoflåt et al., 2017; Unver et al., 2021). The term “international clinical placement” (ICP) or “internship” is also used if the student is undertaking a supervised clinical experience or practicum outside of their original country of study (Browne et al., 2015; Gower et al., 2017; Hilde et al., 2018). In an Australian context, “study abroad” is often used to identify half- or full-year study programs undertaken in different countries in addition to students’ prescribed programs of study (Johnston et al., 2022). The term “international educational experience” is used in this article to describe all types of international programs for nursing and midwifery students.

Previous research around nursing and midwifery international education programs has been undertaken from students’ perspectives, with most studies reporting short-term outcomes only (Bagnasco et al., 2020; Booth & Graves, 2018; Carter et al., 2019; Geraghty et al., 2020). Rarely have any long-term outcomes been investigated and reported (Johnston et al., 2022) indicating a gap in the literature.

The study aimed to determine the long-term outcomes of undertaking an international educational program in a nurse’s or midwife’s pre-registration program on their subsequent nursing and/or midwifery practice. This article presents findings around how participants recognized cultural differences and adapted to them and subsequently how personal and professional practice were informed and developed through participation in such programs.

## Method

### Study Design

A constructivist grounded theory methodology, informed by Charmaz (2006), guided this study. Charmaz (2006) engages a constructivist approach that was considered most suitable to this study as it enabled the researcher and participants to work together to construct meanings in the data, in this case, the impact of undertaking an international educational program during nurse’s or midwife’s pre-registration degree has on their practice. In grounded theory research, data are collected, analyzed, and used to develop a theoretical explanation and generate a theory (Charmaz, 2006). It has been chosen as most appropriate to this study as little is known about the long-term outcomes of international educational programs on nursing and midwifery practice and the generation of theory was the desired outcome (Birks & Mills, 2015). Data were collected through semi-structured interviews with participants, and together with the researcher, the social process of undertaking an international educational program during nurses’ and midwives’ pre-registration education on subsequent professional practice was explored. Approval for the study was granted by La Trobe University Human Research Ethics committee.

### Data Collection

Inclusion criteria required participants to be registered nurse or midwife who had completed at least 1 year of practice and had participated in an international program or placement as a part of their pre-registration degree. Participants were recruited by purposive sampling through advertisement via LinkedIn and Facebook nursing and midwifery groups, followed by snowball sampling as participants passed on study details to fellow nurses and midwives who met the inclusion criteria. Potential participants expressed interest by directly contacting the researcher and provided written consent prior to participation. The interview guide was created specifically for this research study and designed by the research team who have many years of experience in conducting qualitative research. In keeping with Charmaz’s (2006) constructivist grounded theory methodology, the interview guide consisted of open-ended questions, including “How do you relate your experiences of having participated in an international educational program to your current practice?” and “How do you think the international program has influenced or impacted your nursing/midwifery practice?” The interview guide was written in plain English to ensure understanding by participants whose first language may not have been English. Interested participants were interviewed via Zoom for approximately 1 hr each. As interviews took place during the COVID-19 pandemic, the majority of interviews were conducted from the primary researcher’s home office, as work from home was mandated in Victoria, Australia during this time. With participants’ permission, interviews were recorded, later transcribed verbatim, and sent to participants for member checking.

### Data Analysis

In keeping with grounded theory methodology, data collection and analysis occurred simultaneously until categories were saturated. Charmaz (2006) states that grounded theory analysis relies on the constant comparative method, involving ongoing interaction with the data. This approach improves the researcher’s conceptual understanding and guides the analysis process. When compared with other grounded theory approaches, Charmaz (2006) encourages the researcher to reflect on their own assumptions, biases, and interpretations throughout the analysis, allowing for a more reflexive and situated understanding of the data. First, each interview transcript was read line-by-line by the primary researcher to generate initial open and focused codes. Second, codes were compared with new data and emerging ideas. From this process of coding, subcategories and theoretical categories emerged. Initial categories were constantly compared with emerging data and categories were refined as needed (Charmaz, 2006, 2014); In addition, and in keeping with grounded theory methodology, memos were kept as a process of defining meaning and making sense of the data. Analysis resulted in three categories being constructed, *Informing and developing professional practice*, *Recognizing and adapting to cultural differences*, and *Developing as a person*. This article focuses on the category: *Recognizing and adapting to cultural differences.*

## Findings

A total of 22 participants from 8 different countries and 17 different destination countries were included. Nurses and midwives were recruited from eight countries, Australia, England, Scotland, Sweden, Canada, America, Indonesia, and Japan. Among 22 participants, 13 general nurses, two mental health nurses, three midwives, and four dual qualified nurses/midwives participated. The time between the participant’s international educational program to the interview ranged from 2 to 26 years, with the average being 9 years. Most programs were practical (*n* = 8) or clinical placement (*n* = 8), others participated in observational programs (*n* = 5) or theory only (*n* = 5).

The category, *Recognizing and adapting to cultural differences*, describes the process and participants’ application of their cultural learning in professional nursing and/or midwifery practice. This category is further conceptualized through two sub-categories: *Developing cultural safety and cultural awareness* and *Experiencing and adapting to cultural similarities and differences.* The two sub-categories are closely linked as participants developed cultural safety and awareness which led to adaptation of cultural differences and application of learnings in professional practice in the long term.

### Developing Cultural Safety and Cultural Awareness

The sub-category, *Developing cultural safety and cultural awareness*, highlighted that being in a culturally different environment to their own was an important factor in enabling participants’ development of cultural awareness in the context of culturally safe learning environments. Situations where participants could not speak the same language and were simply unfamiliar with daily life were enough to prompt reflection on cultural awareness. One participant who traveled from Canada to Sweden during her undergraduate nursing education on a full semester exchange found the experience helpful in gaining insights into a different culture.

It was a good experience to really dive deep into a different culture . . . it definitely brought in my sense of being aware of different cultures and getting that exposure to being in another culture that you aren’t of yourself, like being a foreigner. That experience of not knowing the language, the experience of learning things for the first time, it was like being an immigrant in another country for a minute. (Participant 5)

Similarly, a nurse from Australia who participated in a nursing exchange program to Sweden in the second year of a Bachelor of Nursing degree felt the program facilitated improvement in communication with patients from a non-English-speaking background. Improved communication skills led to increased cultural awareness and ultimately improved cultural safety as these skills were embedded in her nursing practice in the long term.

*. . . those cultural awareness and communication skills definitely improved after* going on exchange . . . . that still applies with patients today, whenever I’m with a patient from a non -English speaking background, being able to build trust and having the patient trust you as a nurse and being able to reassure them, even if you’re not speaking the same language. And I’ve had so many patients where that’s been the case and so it’s made me more comfortable with nursing patients from different backgrounds. (Participant 2)

Being supported to learn in a culturally safe manner was important and involved time for reflection, challenging own behaviors and ways of thinking. For some, this meant working with the host community to truly understand the needs of the local community. An Australian midwife who participated in a short-term program to Vanuatu during their final year of Bachelor of Midwifery found:

Time was given to learn in a culturally safe manner . . . really challenging our assumptions around knowledge was probably when I started to think more critically about it . . . as far as traditional ways of knowing that people may bring to their healthcare interactions that the western paradigm is going to diminish or decide to rule out as inappropriate . . ., certainly there’s still traditional birth attendants working in Vanuatu and so honouring and understanding the immense historical knowledge. (Participant 1)

Similarly, an Australian nurse who participated in a short-term program in Northern Thailand over 20 years prior to her undergraduate degree reported that cultural safety meant having mutual respect between participants on the program and host community. The long-term outcome of being changed as a person and impact on professional practice was evident:

We got to learn a lot off them (the local nurses), and they got to learn off us. And it wasn’t about changing ways or anything like that, it was a mutual respect of what we both could bring to the table . . . it’s changed me, that has brought me a better awareness with culture. It changed the way I look at our health care system and it also made me look at patients’ health literacy, for example, I might seek an interpreter or get a family member in who’s more proficient in English that they can interpret for the patient. (Participant 8)

Similarly, the process of cultural learning and the participant’s development in relation to culturally congruent health care was evident through her reflection on how the experience had a long-term influence on her nursing practice.

It made me more culturally aware of the various cultures that we do nurse . . . this is becoming a bigger thing now, having a cultural awareness and I get it all now. The different people that we have coming through, Australia is very multicultural society, so it was about a bigger cultural awareness for me in dealing with different patients and I think it was gratitude was the biggest thing- how lucky we are . . . I’m more culturally aware and understanding and I suppose having that empathy of what they’re going through and trying to seek out more support for them or whatever they need. (Participant 8)

Long-term outcomes were evident for a registered nurse from the United Kingdom who participated in a 4-week program to Cape Town, South Africa, who found the cultural understanding gained had an important impact on her subsequent nursing career as she was able to implement a change in practice.

The cultural understanding (I gained) could be the biggest influence on my nursing career because you simply can’t teach that (in the classroom). It has given me a depth of understanding regarding cultural differences, such as food preferences, family involvement and family hierarchy. As a nurse, I make sure I show an understanding and make allowances to be able to engage with patients and their families more effectively. (Participant 19)

### Experiencing and Adapting to Cultural Similarities and Differences

The sub-category, *Experiencing and adapting to cultural similarities and differences*, highlights how the experience of participation in a program was both informative and often transformative for participants’ nursing and midwifery clinical practice. The process of cultural learning took place across the span of participants’ programs and was not simply one experience or one moment in time. The experience of being in unfamiliar environments and cultures was a significant learning curve and contributed to participants’ development and application of learning in relation to providing culturally congruent health care. A Canadian registered nurse who traveled to Sweden on exchange during her Bachelor of Nursing felt that being in-country was necessary to ensure cultural learning and was something that could not be learned in the traditional classroom.

Having that cultural dissonance, trying to fit into a different culture is something that can’t be learnt out of a textbook. You have to live it to really understand it. I think that was a really valuable life skill, not just with my nursing career, but putting myself in somebody else’s shoes for a minute and having that empathy is something that I’ve definitely come back to time and time again and definitely with regards to my nursing practice. (Participant 5)

However, it was not just cultural differences of the countries they visited that participants needed to adapt to. For some, the cultural differences of their peers also contributed to cultural learning.

We were in a different country at the age of 20, learning how to interact with others. I didn’t speak the language . . . . we were staying in very multi-cultural accommodation and lots of people from all over Europe, America, Australia living there . . . it just gave me a perspective of underlying currents and culture, as much as you want to say it doesn’t exist, that there is a racial divide. And that was very interesting because as a young person, you don’t understand, it is only when I thought about it several years later, and it has definitely influenced my way of thinking. (Participant 19)

The impact on practice for participants was a changed perspective and improved cultural awareness “It changes your whole perspective and the way you think about things. . . . .you need to be aware that there are differences between cultures and to make allowances for that” (Participant 19). Considering people’s cultural preferences was noted to be important and could be translated into practice by considering patient’s food preferences as it was recognized that “food makes a really big difference for people when they’re in hospital” (Participant 4).

An Australian registered nurse working in the specialty of patient pain management at the time of interview, and who participated in a short-term program to Vanuatu, found she was able to compare and contrast patients’ expressions of pain from a cultural perspective. This outcome was translated to the participant’s current practice through improved pain assessment skills and management.

One of the key things from the program was seeing the significant cultural difference in the way people express pain. We see (in current practice) patients experiencing and expressing pain in different ways and often get reports of patients that doesn’t look like they’re in pain, but just because they don’t say it, that doesn’t mean that they’re not experiencing it. I think that has probably been something specific to my current practice. (Participant 4)

The experience of cultural learning was transformative for some as a change in thinking occurred which then translated into a /shift in practice.

It’s a different culture. You know, you have to be very aware of that and link in some of these different cultural practices to what’s going on. it was a lot of challenging our thinking and mind shift which was just mind blowing . . . . we go out of our way so that our patients are informed and know what’s going on. I put it in a way that they are able to understand. It is something that I developed through my placement overseas, but it is also a way that I am changing in my practice these days. (Participant 8)

Experiencing traditional customs in Vanuatu brought heightened cultural awareness in the long term for a registered nurse who traveled from Australia to Vanuatu to participate in a short-term global health program. Heightened cultural awareness translated into her nursing practice and resulted in a greater understanding and appreciation as to why patients may or may not accept health care and is still relevant 19 years post participation in the program.

There was a certain number of people that still have some beliefs in what they call “kustom medicine” which is actually practised by what I would call witch doctors . . . so I guess it brings in awareness of other people’s cultural or religious beliefs and their acceptance of health care. (Participant 11)

A mental health registered nurse from Scotland who participated in a program in Vietnam reported developing cultural awareness skills for the first time as her home country was not diverse in religion or culture.

Being from Scotland, I had never come across patients of different religions and cultures. (Participant 15)

The long term impact was a shift in practice in assessment and management of mental health patients in her care due to a heightened appreciation of the importance of religion and culture on a person’s mental health, “a patient’s religion and culture may impact on them mentally and explain where some of their family pressures arise” (Participant 15).

A registered midwife who traveled from Australia to Uganda on a 2-week global learning program, spent 1 week working clinically in a tertiary hospital in Uganda followed by another week participating in a community engagement experience found that the experience in Africa had long-term impact on her midwifery practice as she was able to apply learnings to her day-to-day patient interactions.

I feel like what it actually changed for me is to really set me on a path towards understanding health literacy. After working for several years now in a very busy, very diverse public hospital, I really experience on a day to day basis, is that you can’t assume about what someone might know. I think my experience in Africa just really opened my eyes in that way, how do you decide where you’re going to pitch antenatal education, making an assumption that someone knows (female anatomy). It’s not an assumption that I would make now. I think that I’m a lot more careful when I talk to families to really listen and try and understand what they want to know and what’s important to them to know. (Participant 3)

A Swedish midwife who participated in a clinical placement in Australia described the cultural awareness gained from the experience and importantly how these skills had since translated into her midwifery practice 3 years since her participation.

The program gave me the chance to see different cultures and has affected me when I meet patients now that are from different cultures, I ask them about their culture and if there is anything culturally specific they require, (it is now) easier for me to help patients in that way. (Participant 16)

Similarly, a registered nurse from the United States who participated in an art tour to France as an elective in her nursing degree described how although the program was not directly related to health care, it had informed and shifted her nursing clinical practice in a positive way.

I totally see the correlation between that trip and my future experiences and I can absolutely see how it’s informed, even without the experience of going into a hospital, I can see how learning about culture lent my mind to things that I applied when I was a student in clinical and then later in my bedside practice because I learned about a culture that I had never been exposed to before, I had the experience of going and not speaking the language and struggling. I took those lessons with me and was able to absorb them into clinical practice, a really specific example is when I think about family involvement in care and seeing a very family -oriented culture and see family member involvement in France. I think it really opened my eyes to understanding the advantages that the patients that I was caring for, the ones that came in with family and support structures, really had such an advantage compared to my patients who were coming in and maybe didn’t have those. I don’t know if I realized it at the time but understanding deep down that I had to advocate on their behalf because maybe connections to resources may have been missing. (Participant 6)

## Discussion

This study sought to explain how nursing and midwifery professional practice was impacted through participation in international educational programs. The category, *Recognizing and adapting to cultural differences*, which was further conceptualized through two subcategories: *Developing cultural safety and cultural awareness* and *Experiencing and adapting to cultural similarities and differences* describes the processes of participants’ cultural learning and application of the learning in relation to culturally congruent health care.

The sub-category, *Developing cultural safety and cultural awareness*, describes participants’ experiences of recognizing and experiencing a culture different from their own. Learning that subsequently occurred related to their experiences needed a culturally safe space for learning and led to improved cultural awareness. Cultural learning described in this study is similar to that in previous studies; however, adds an important key understanding, namely that benefits are maintained over time and continue to impact a nurse’s or midwife’s professional practice contributing to culturally safe, quality patient care. Watson (2015) investigated the influence of an international clinical experience during baccalaureate education on interprofessional collaboration and teamwork among newly qualified registered nurses from the United States within their first year of employment. Findings revealed seven themes, with the theme “Practicing cultural sensitivity and awareness” most relevant to the current study, as participants noted that they practiced nursing through a “cultural sensitivity lens by being less judgmental” (p. 88).

The cultural awareness gained by nurses and midwives in the current study led to the adaptation of cultural differences, and new learnings were embedded into their nursing and/or midwifery practice. A recent review (Johnston et al., 2022) found a majority of the 56 included studies described outcomes related to students’ attainment of cultural learning in the short term. Booth and Graves (2018) reported that students progressed toward cultural competence and global awareness. In other studies, students described developing cultural sensitivity, cultural knowledge, and cultural skills (Meaux et al., 2021). The range of benefits for students varied in Nourse’s (2022) study and included cultural awareness, as well as changed perspectives. Students challenged their beliefs about culture and through authentic experiences, reported outcomes were increased awareness of community needs, decreased stereotyping, and increased confidence in working with culturally diverse populations (Booth & Graves, 2018). Other research studies have demonstrated that future nurses’ preparedness for culturally competent nursing care developed during their international programs, including enhancing cultural understandings (Kohlbry, 2016; Trapani & Cassar, 2020).

*Experiencing and adapting to cultural similarities and differences* explained the attainment of cultural learning and how knowledge was applied to professional nursing and/or midwifery practice in the long term. For some participants, programs influenced how they practiced and continued to influence their clinical practice. Very few previous studies have focused on long-term outcomes, with the majority conducted within 6 months post-program and focused on students’ experiences. Only one study (Ulvund et al., 2023) was found that investigated eight nurses’ experience who participated in an international clinical placement in Ethiopia during their pre-registration nursing degree at one university in Norway. Participants completed their program 2 to 8 years prior to the interview and findings found that the international clinical placement increased participants’ global and cultural competence which was of ongoing benefit in their nursing practice (Ulvund et al., 2023).

Findings from this current study build upon the existing body of work regarding the cultural benefits of international educational programs on professional nursing and midwifery practice. However, this study adds new understandings about the benefits of international educational programs, particularly, how cultural learnings are embedded and continue to inform one’s nursing and midwifery professional practice in the long term. In the case of some participants in this study, benefits on practice were ongoing, some being more than 20 years or more post-program. These findings have broad implications for industry, especially for education providers and employers. Long-term impact on nurses and midwives shows the attainment of cultural skills that have benefits for culturally safe, quality patient care.

The need to provide a range of international educational programs to nursing and midwifery students is paramount so students can develop their cultural humility and global citizenship attributes and apply the learned knowledge and skills in their practice. Nursing and midwifery curriculum providers must be supported in facilitating culturally appropriate international educational programs which should be conducted in a culturally safe space for student learning ensuring students are adequately prepared to reflect and learn. The cultural learnings gained from international educational programs are of benefit to the nurses and midwives who participated but also the patients they subsequently care for by promoting culturally congruent health care. The positive and ongoing influence on one’s professional practice is important for employers who require their staff to be culturally safe practitioners. Nurses and midwives who have participated in international educational programs are likely to be culturally humble, global citizens which are important qualities for those wishing to pursue leadership opportunities (Campos-Moreira et al., 2020). Therefore, it is important for employers to be aware of staff who have participated in cultural learning programs when workforce planning.

This study has highlighted the long-term benefits of participation in an international educational program; however, participation is acknowledged to be difficult for some students often due to financial costs and caring responsibilities at home. Students who are unable to leave their home university may benefit from a virtual program. Internationalization at home and Collaborative Online International Learning (COIL) programs have gained popularity and offer students an intercultural experience without leaving their home university (Johnston & Malik, 2020). These programs are emerging as a cost-effective pedagogy as a way to prepare nursing and midwifery students to work in multi-cultural health care settings (Malik & Johnston, 2022).

This study does have some acknowledged limitations. The methodology used meant the findings could not be generalized. However, the inclusion of participants from a range of countries who traveled to a range of destinations provides wide perspectives, although may not be representative of all cultural backgrounds. Only nurses and midwives who could speak and understand English were interviewed, hence the experiences of non-English-speaking nurses and midwives may be different. Further research offering insights into those experiences from non-English-speaking nurses and midwives, and from different regions of the world, participating in international educational programs during their pre-registration studies is recommended. Furthermore, it is recommended to follow up with employers regarding the practice of those staff who have participated in cultural learning experiences and how their learnings can be optimized in the clinical setting.

## Conclusion

This study provides knowledge of the long-term outcomes of undertaking international mobility programs during nursing or midwifery pre-registration education. This paper focused on how cultural skills and professional practice for nurses and midwives were informed and developed through participation in international educational programs. This is an important contributor to the provision of culturally congruent health care. Findings from this study support the ongoing need for institutions to provide nursing and midwifery students with opportunities to participate in such program during pre-registration education. Positive and ongoing influences on one’s professional practice are critical for employers in workforce planning and particularly relevant as a means to promote cultural safety and quality patient care.

## Implications for Culturally Congruent Health care

- International educational programs have long-term benefits for participants, which is transferred into their professional nursing and midwifery practice.
- Long-term impact on nurses and midwives shows the attainment of cultural skills that has benefits for culturally safe, quality patient care.
- Informs nursing and midwifery curriculum globally that expose students to various cultures through international educational programs.
